# Dynamic Change and Target Prediction of Axon-Specific MicroRNAs in Regenerating Sciatic Nerve

**DOI:** 10.1371/journal.pone.0137461

**Published:** 2015-09-02

**Authors:** Monichan Phay, Hak Hee Kim, Soonmoon Yoo

**Affiliations:** 1 Nemours Biomedical Research, Alfred I DuPont Hospital for Children, Wilmington, Delaware, United States of America; 2 Department of Biological Sciences, University of Delaware, Delaware, Newark, United States of America; Hertie Institute for Clinical Brain Research, University of Tuebingen., GERMANY

## Abstract

Injury to axons in the peripheral nervous system induces rapid and local regenerative responses to form a new growth cone, and to generate a retrogradely transporting injury signal. The evidence for essential roles of intra-axonal protein synthesis during regeneration is now compelling. MicroRNA (miRNA) has recently been recognized as a prominent player in post-transcriptional regulation of axonal protein synthesis. Here, we directly contrast temporal changes of miRNA levels in the sciatic nerve following injury, as compared to those in an uninjured nerve using deep sequencing. Small RNAs (<200 nucleotides in length) were fractionated from the proximal nerve stumps to improve the representation of differential miRNA levels. Of 141 axoplasmic miRNAs annotated, 63 rat miRNAs showed significantly differential levels at five time points following injury, compared to an uninjured nerve. The differential changes in miRNA levels responding to injury were processed for hierarchical clustering analyses, and used to predict target mRNAs by Targetscan and miRanda. By overlapping these predicted targets with 2,924 axonally localizing transcripts previously reported, the overlapping set of 214 transcripts was further analyzed by the Gene Ontology enrichment and Ingenuity Pathway Analyses. These results suggest the possibility that the potential targets for these miRNAs play key roles in numerous neurological functions involved in ER stress response, cytoskeleton dynamics, vesicle formation, and neuro-degeneration and-regeneration. Finally, our results suggest that miRNAs could play a direct role in regenerative response and may be manipulated to promote regenerative ability of injured nerves.

## Introduction

Axonal injury to the peripheral nervous system (PNS) triggers active translation of the localized transcripts that have been transported into axons from the cell body [1–4]. Intra-axonal translation of the mRNAs has been directly linked to spontaneous regenerative responses of PNS neurons [5–11]. For example, PNS nerve injury triggers a rapid translation of importin-β1, vimentin, RanBP1, and Stat3 mRNAs in axons [5, 8–10, 12]. The proteins encoded by these axonal mRNAs provide for retrograde injury signals to the cell body. Furthermore, Verma et al [13] has shown that axonally synthesized proteins are needed for the reformation of growth cones, which are critical for subsequent regeneration of injured axons. These localized mRNAs are translationally dormant in the axon until recruited into translation machinery following injury. However, it is unclear how the activated local translation following injury becomes silent when it is no longer needed.

MicroRNAs (miRNAs) are a class of small regulatory non-coding RNAs (~22 nt long). They have been proposed to negatively regulate translation in the nervous system at the posttranscriptional level in a sequence-specific manner [14–20]. Recent evidence shows numerous miRNAs in the mammalian central nervous system (CNS) and PNS neurons, suggesting that they could play important roles as molecular switches to alter expression of protein cohorts through binding to multiple mRNAs in neurons [21–23]. Interestingly, several groups have further shown temporal changes of miRNA expression in the spinal cord, as well as in the sciatic nerve following injury [20, 24–26]. These studies suggest direct roles of miRNAs in nerve regeneration via regulating protein synthesis.

Although a recent study reported altered levels of miRNA in the proximal stumps of the sciatic nerve to the injury site at five post-injury time points [26], a big question still remains when it comes to axon-specific miRNAs directly responsible for the involvement in regenerative responses to injury. The contamination from non-neuronal cells during microdissection of nerve tissues and axonal RNA isolation from whole nerve lysate (axoplasm) possibly argue that the RNA impurities may have impacted on the subsequent bioinformatics analyses for the differential miRNA expression profiling, leading to errors in analytical measurement results. Here, we attempt to purify axoplasmic lysate from the proximal stump of the sciatic nerve to generate new insight into temporal responses of axon-specific miRNAs in response to injury through deep sequencing studies.

We find that axoplasmic miRNA levels in rat sciatic nerve are altered, upon injury. We identify a total of 141 rat miRNAs from the proximal stump of the sciatic nerve to the injury site. We further focus on bioinformatic analyses of seven most up-regulated and eight most down-regulated miRNAs with at least a mean fold change of 2 in regenerating nerve, compared to those in naïve nerve. By integrating predicted potential target mRNAs and 2,924 transcripts known to be localized to distal axons of DRG neurons [27], we suggest the possibility that the targets of the intra-axonal miRNAs are directly related to multiple biological and neurological functions including regenerative responses following injury.

## Materials and Methods

### Animal Surgery and Tissue Preparation

Animal procedures were approved (Approved protocol number NBR-2014-001) by the Institutional Animal Care and Use Committees (IACUC), and the experiments were conducted under the IACUC at Alfred I. DuPont Hospital for Children. For injury, 150–225 g male Sprague Dawley rats were deeply anesthetized using intraperitoneal injection of ketamine (50 mg/kg) and xylazine (10 mg/kg). Once completely anesthetized, a small incision (approximately 0.5 cm) was made over the sciatic nerve at the mid-thigh level. The sciatic nerves were surgically exposed by gentle separation of the quadriceps muscle and manually crushed using fine jewelers’ forceps twice for 15 seconds each. After closing the incisions with 4·0 Ethilon sutures, animals were returned to cages and observed for the degree of nerve crush injury as evidenced by contracted hind paw. From closely observing animals with peripheral nerve injury, they experienced little pain or discomfort after the nerve crush procedure. To minimize possible pain and distress, animals were visually checked daily for any signs of systemic or localized infection (e.g., indurations or weeping of clear or cloudy fluid) as well as any evidence of pain or distress until sacrifice for the study. Any complications including gnawing at the hindlimb or incision site or open incision, excessive indurations of the incision, weight loss or lack of grooming were recognized as the evidence of pain or distress. Although we had only rarely seen any complications, animals that exhibited these features were euthanized and carcasses disposed by the regulations.

To evaluate regenerative response of nerves to injury *in vivo*, animals were euthanized at 1, 4, 7, and 14 days post-injury (PDI) by asphyxiation with CO_2_ using compressed sources of gas, and sciatic nerves ipsilateral (injured) and contralateral (uninjured) to crush injury were collected and processed as previously described [28].

### RNA Extraction and Illumina Sequencing

To isolate total RNA from whole sciatic nerves, we utilized the mirVana™ miRNA Isolation Kit (Invitrogen) following the manufacturer’s instructions. For comparison purposes, a mechanical squeezing method was used to isolate total RNA from nerves as previously described with a few modifications [29]. After determining RNA yield by fluorimetry using *RiboGreen* (Invitrogen), 100 ng RNA was used to reverse transcribe to cDNA using *iScript RT kit* (BioRad) and subsequently carried out for standard extended PCR (35 cycles). β-actin, cell body-restricted [microtubule-associated protein 2 (MAP2) and H1 histone family member 0 (H1F0)], and non-neuronal cell [glial fibrillary acidic protein (GFAP) and Receptor tyrosine-protein kinase ErbB family-3 (ErbB-3)] mRNAs were used to assess purity of RNA isolation. Purified RNA was further fractionated to <200 nucleotides in length by using an *RNeasy Mini spin column* followed by the *RNeasy MinElute Cleanup Kit* (Qiagen).

For small RNA library construction and deep sequencing, a total of 15 samples of sciatic nerves collected at different time points (1, 4, 7, and 14 DPI) including uninjured nerves (n = 3/each time points) were subjected to Illumina HiSeq 2500 sequencing by the University of Delaware DNA Sequencing and Genotyping Center (UDSGC) located in the Delaware Biotechnology Institute (DBI) in Newark, DE. In brief, adapters were ligated to the 5’ and 3’ termini of the purified small RNAs followed by cDNA synthesis (llumina TruSeq Small RNA Library prep kit). The resultant cDNA was PCR amplified using a common primer and Illumina index primer for the purpose of multiplexing in a single flow cell on Illumina HiSeq 2500. Resulting sequence data were analyzed by the CLC Genomics Workbench (Qiagen) software package with support from the University of Delaware Center for Bioinformatics and Computational Biology (CBCB) Bioinformatics Core. After trimming off adapters and counting reads, length distribution was calculated and filtered to partition miRNA from other small non-coding RNAs. Trimmed sequences ranging from 16–25 nt were annotated to rat miRBase 21 and sequences ranging from 23–36 nt were annotated to piRNABank rat database. Only sequences containing 50 reads or greater were use for differential expression analysis.

### Clustering methods

To assess the relationship between significantly altered miRNAs in response to injury, we used hierarchical clustering method using *Cluster 3*.*0* open source clustering software. The similarity metrics across different time points after injury including uninjured control were calculated using average linkage and Euclidean distances metric.

### Validation of miRNA alterations by quantitative real time PCR (qPCR)

To validate Illumina deep sequencing data, we determined the profiles of miRNAs by quantitative PCR (qPCR) analysis using miRNA-specific LNA primers (Exiqon). In brief, the purified small RNA was converted to cDNA using *NCode* ***™*** *VILO* ***™*** *miRNA cDNA Synthesis Kit* (Invitrogen) to optimize reverse transcription efficiency from the small RNAs. The resulting cDNA was then used as a template for qPCR analyses using *NCode* ***™*** *EXPRESS* ^***®***^ *SYBR GreenER* ***™*** *miRNA qRT-PCR Kit* (Invitrogen) on the ABI Prism 7900HT. We initially evaluated whether the most common reference RNAs used in qPCR experiments, such as 5S and 12S rRNAs, tRNA, and U6 snRNA, showed uniform level across the samples by plotting the raw C_T_ values for each samples. Our variance analysis in expression levels of each of the reference RNAs showed that U6 snRNA was the most consistently expressed RNA, followed by tRNA, 12S, and 5S rRNAs. Thus, the relative level of miRNAs was normalized to that of U6. The final results of qPCR in relative level were expressed as the ratio of miRNA in the injured nerve to that in the uninjured nerve using the 2^−ΔΔCT^ method. To verify miRNA-specific primer specificity, we confirmed a single peak in the melting-curve analysis, as well as run the reaction products on a 5% agarose gel (NuSieve 3: 1; Lonza) for all genes analyzed.

### miRNA Target prediction

A combinatorial strategy was used to predict target mRNAs for the differentially expressed miRNAs using two algorithms, TargetScan and miRanda. The target mRNAs commonly predicted by these algorithms were then overlapped with a dataset that previously identified axonally localized transcripts [27] to identify intersected target mRNAs.

### Functional annotation analysis of the predicted targets

Gene Ontology (GO) enrichment analysis was used to identify biological functions that were most significant to the predicted axonal target of each significantly altered miRNA following injury.

Ingenuity Pathway Analysis (IPA) (Ingenuity System Inc.) was used to identify the significant pathways in the nervous system for the predicted intra-axonal target mRNAs according to the KEGG database. The significance was measure by the number of predicted molecules that map to the pathways and a *p* value that determine the probability of the association between the predicted molecules and the pathway.

### Statistical analysis

Differences in altered miRNA levels across different time points after injury was assessed using the Chi-squared test, and the false discovery rate (FDR) was calculated to correct the *p* values; the smaller FDR indicates the smaller error in judging the *p* value. *p* values <0.05 were considered statistically significant. The *edgeR* program was used for all statistical analyses.

## Results and Discussion

### Preparation of miRNA-enriched small non-coding RNA samples and Illumina small RNA sequencing

A similar study recently identified 225 known miRNAs from rat sciatic nerve and 201 miRNAs from DRG neurons showing significant changes in levels at five time points after nerve injury [26]. However, 36 of 225 significantly altered miRNAs found in the sciatic nerve did not overlap with those found in the cell body. This argues that the results could have been severely dampened by the fact that the small RNAs prepared from the whole sciatic nerve included not only nerve axoplasmic RNAs, but also other RNAs from non-neuronal cells. Given that contamination from non-neuronal cells, particularly Schwann cells, is a widespread problem in axoplasm preparations from the sciatic nerve, the significant biases might have been introduced to the data analyses, as the majority of cellular miRNAs are present in both the nucleus and the cytoplasm within the cell body [30, 31]. In addition, since some miRNAs can exhibit differential expression patterns in different cell and tissue types, Yu et al. study [26] may not have provided precise insights for nerve-specific response at the intra-axonal level of miRNAs to injury. This could hamper our efforts to develop new therapeutic strategies for improving nerve regeneration. Therefore, our approach to purify small RNAs from sciatic nerve axoplasm will not only reduce the biases that the previous report might have introduced, but also improve the apparent representation of differential axonal miRNA levels in regenerating nerves.

**Fig 1:**
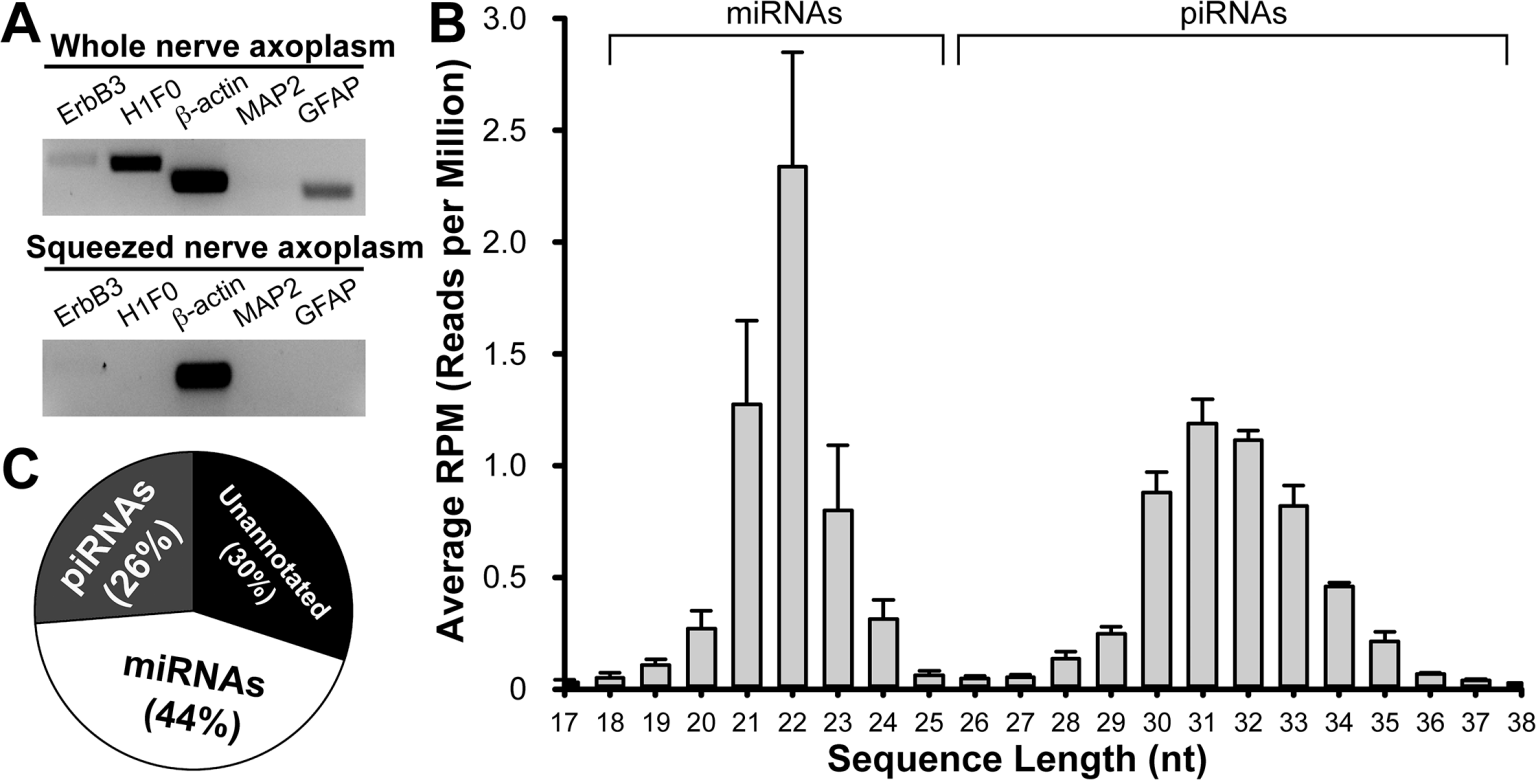
Enrichment and detection of small non-coding miRNA from sciatic nerve axoplasm. **A.** Extended RT-PCR of mRNAs from sciatic nerve axoplasm using either by the conventional whole tissue lysate method or a mechanical squeezing procedure. Note that neither cell body restricted (MAP2, H1F0) nor glial (GFAP, ErbB3) mRNAs were amplified from sciatic nerve cDNAs reverse-transcribed from the squeezed axoplasmic RNAs. **B.** Size and frequency distribution of regulatory and small non-coding RNAs in uninjured sciatic nerve. **C.** A size histogram of mapped small RNAs. >40% of small RNAs were mapped to miRNAs, while 30% of small RNA sequences could not be annotated.

We first assessed the purity of axoplasmic RNAs extracted from the proximal stumps of the sciatic nerve. The conventional method for extracting axoplasmic RNA in the previous study was based on whole nerve axoplasm of dissected sciatic nerve segments [26]. To directly compare the purity of axoplasmic RNAs extracted at different days post-injury (DPI), we standardized the extraction of the nerve to the 10-mm segments located 3–5 mm proximal to the injury site (ipsilateral) and the corresponding uninjured nerve (contralateral) excluding the site of injury, which reduced non-neuronal contamination of the preparations. We examined the purity of axoplasmic RNAs extracted from the whole axoplasm using extended cycle RT-PCR with gene-specific primers for cell body restricted [microtubule-associated protein 2 (MAP2) and H1 histone family member 0 (H1F0)] and glial cell [glial fibrillary acidic protein (GFAP) and Receptor tyrosine-protein kinase ErbB family-3 (ErbB3)] mRNAs. The results showed significant levels of glial contamination in the axoplasmic lysate (Fig 1A). To isolate purer axoplasmic RNAs, we next optimized the mechanical squeezing procedure that the Fainzilber group has developed [29, 32]. Briefly, the sciatic nerve segments were manually squeezed with a plastic pestle that fits Eppendorf tubes on ice in the Lysis buffer, followed by precipitating RNA with ethanol according to the mirVana™ miRNA Isolation Kit. The extended cycle RT-PCR showed undetectable levels of MAP2 and H1F0) and GFAP and ErbB3 mRNAs in the axoplasmic lysate, suggesting the very low levels of non-neuronal cell contamination in axoplasmic RNAs extracted from the squeezing procedure in comparison with the conventional whole nerve extraction procedures (Fig 1A). To compare dynamic alterations of miRNAs in sciatic nerve following injury, small RNA libraries were generated at different DPIs after fractionating small RNAs [<200 nucleotides (nt) in length] using an RNeasy Mini spin column followed by the RNeasy MinElute Cleanup Kit.

After trimming off adapter sequences from the raw sequencing data of Illumina HiSeq 2500, a dataset of 10.9 ± 1.5 (uninjured), 12.4 ± 1.0 (1 DPI), 11.0 ± 0.8 (4 DPI), 11.4 ± 1.1 (7 DPI), and 10.1 ± 1.5 (14 DPI) million reads, ranging from 17 to 38 nt, was obtained (Fig 1B). Further examination of these small RNA sequences revealed that 43.6% ± 8.1, 46.7% ± 4.4, 57.6% ± 2.5, 40.0% ± 2.4, and 32.1% ± 2.1, respectively, of these reads were mapped to miRNAs, with a relatively minority of the small RNAs being annotated as piRNAs (26.6% ± 2.7, 20.4% ± 1.8, 15.0% ± 1.4, 28.6% ± 1.3, and 33.4% ± 2.1, respectively) (Fig 1C). The size distribution of the mapped miRNAs showed a peak at 22 nucleotides and almost half of these reads (44.9%, 41.6%, 37.1%, 38.4%, and 40.7%, respectively) were 22 nucleotides in length, consistent with the size criteria of miRNA (Fig 1B). By mapping to the rat miRBase 21, we were able to annotate a total of 141 rat miRNAs with average read counts greater than 50 (S1 Table).

### Temporal changes in miRNA levels in the regenerating sciatic nerve

**Fig 2:**
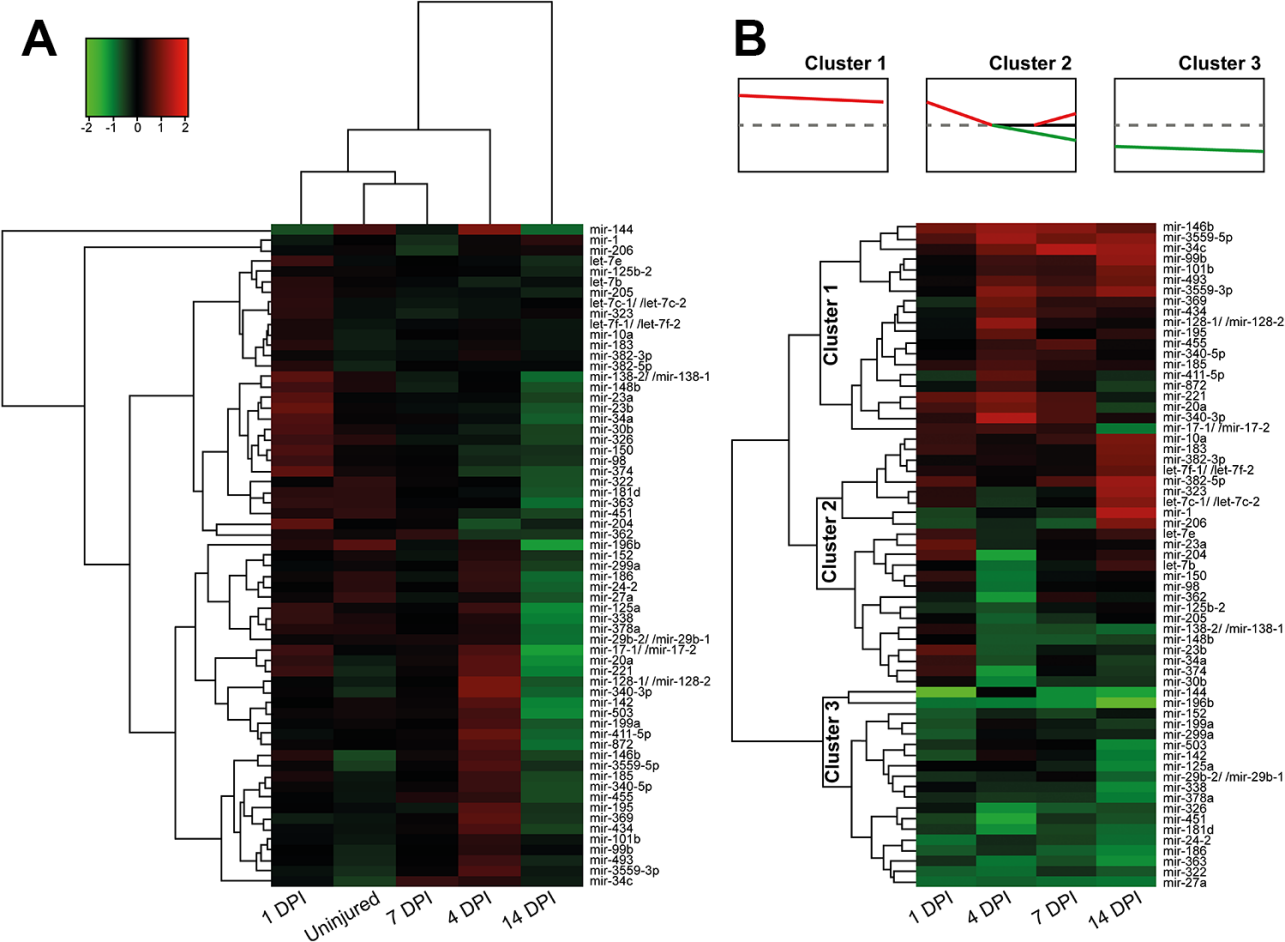
Distinct pattern of miRNA levels in sciatic nerve in response to injury. The heatmap with a cluster dendrogram showed significant changes in levels of 63 miRNAs in rat sciatic nerve that received a crush injury and were sacrificed at 1, 4, 7, and 14 days post-injury (DPI), as compared to the sham-operated uninjured control (n = 3/group). **A.** The color scale shown on the top left denotes the relative expression level of the indicated miRNA across all time points (log_2_ scale): red represents an increased change in level and green denotes a decreased level. Clustering analysis was performed using *Cluster 3*.*0* with an average linkage and Euclidean distances metric and visualized using Java *TreeView*. **B.** Each column represents different time points after injury [1, 4, 7, and 14 days post-injury (DPI)], as compared to the uninjured control (n = 3/group), and rows represents individual miRNAs. Red represents an increased change in level compared to that in the uninjured control and green denotes a decreased level. Insets on the top show three distinct clusters of miRNA changes. Dashed lines indicate miRNA levels in the uninjured control.

To identify miRNAs whose axoplasmic levels are significantly changed following injury, the annotated axoplasmic miRNAs were subjected to statistical analysis using the *edgeR* program. Of 141 annotated axoplasmic miRNAs, the statistical analysis revealed 63 rat miRNAs that were differentially regulated with different false detection rate (FDR) between the contralateral uninjured nerve and the ipsilateral injured nerve at any time points after injury (p<0.05 and FDR<0.05) (Fig 2A). Cluster analyses were carried out to further determine if the axoplasmic levels of these 63 miRNAs could be distinguished between these groups. To this end, first, the dataset from each time point including the control was imported into *Cluster 3*.*0* open source clustering software, and calculated the similarity metrics using average linkage and Euclidean distances metric. Fig 2A visualized the results of hierarchical cluster analysis in the heatmap. The hierarchical clustering was based on the assumption that changes in axoplasmic miRNA levels would correlate with the time course of the regenerative responses occurring in the PNS axon regrowth; for example, miRNAs clustering closest to each other should be those involved in nerve regenerative processes, presumably peak at 7 DPI in a rat sciatic nerve injury model. In a previous study, Yu et al. [26] reported that miRNAs from sciatic nerves at 4, 7, and 14 DPI were clustered together and separate from the cluster of the control and injured nerves at 1 DPI, supporting the notion that the level of small RNAs seems fit the time course of regenerating sensory axons. However, inconsistent with this previous study, our clustering results showed that miRNAs from the injured nerve at 14 DPI were not clustered together with those from injured nerves at 4 and 7 DPI. Rather, the uninjured control and injured nerves at 7 DPI were clustered together close to that of an injured nerve at 1 DPI, suggesting that it did not follow such a simple assumption shown in the previous study. This argued that the temporal alterations of miRNA levels in the sciatic nerve following injury are not a simple reflection of regenerative responses, but rather an intricately complex and balanced mechanism by which post-injury responses are precisely controlled.

To cluster axoplasmic miRNAs by their temporal changes with similar responses to nerve injury, the datasets from each time point were directly compared with the uninjured control and the mean fold-change values were calculated for hierarchical cluster. As shown in Fig 2B, the analysis classified these miRNAs into three distinct clusters. One cluster near the top of the heatmap showed miRNAs that significantly increased along the time series. A second cluster contained miRNAs that first increased at 1 DPI and then decreased. A third cluster near the bottom of the dendrogram represented miRNAs that significantly decreased along the time series after injury. These results suggest that individual axonal miRNAs in sciatic nerve may play different important regulatory roles in local mRNA translation at different times following injury.

### Validation of RNA sequencing data using Q-PCR

**Fig 3:**
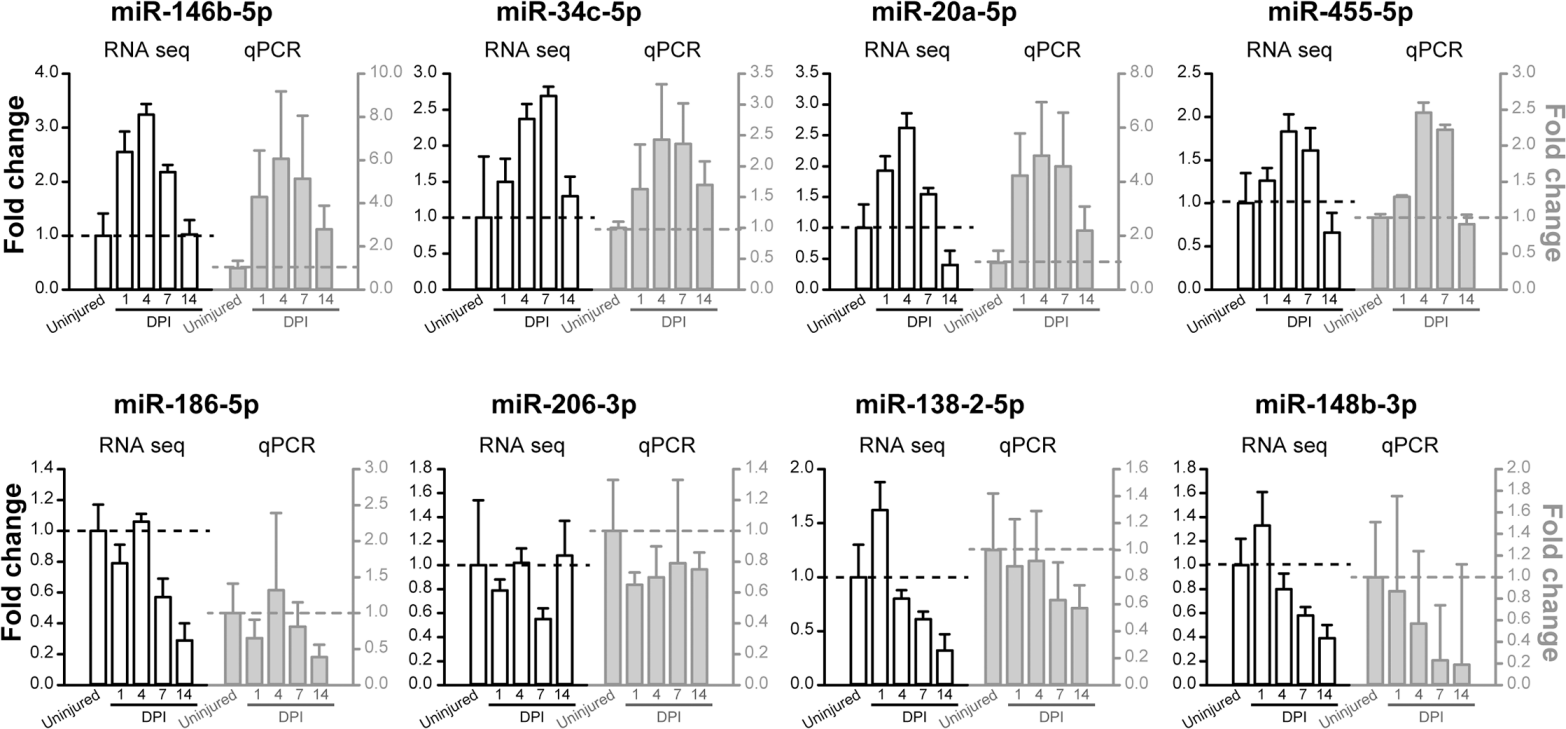
Validation of mature miRNA sequencing data by real time qPCR assay. Bar graph showed changes in levels of 8 miRNAs at 1, 4, 7, and 14 days post-injury (DPI), as compared to those of uninjured control. Top panels. miRNAs significantly up-regulated following injury. Bottom panels. miRNAs significantly down-regulated following injury. Error bars indicate standard deviation.

To verify Illumina RNA sequencing data, we examined the axoplasmic level of a subset of miRNAs including four up-regulated (miRs-146b-5p, -34c-5p, -20a-5p, and-455-5p) and four down-regulated miRNAs (miRs-186-5p, -206-3p, -138-2-5p, and-148b-3p) by qPCR using miRNA-specific primers (Table 1 and Fig 3). These studies showed a strong correlation between our RNA sequencing data and qPCR data, suggesting that Illumina RNA sequencing data accurately reflected the molecular changes in the sciatic nerve following injury and were reliable to warrant further analysis.

**Table 1:** Seven miRNAs significantly up-regulated and eight miRNAs down-regulated following nerve injury.

		P value	FDR
**Up-regulated miRNAs**			
	miR-146b-5p	2.75E-07	4.16E-05
	miR-20a-5p	8.47E-04	2.03E-02
	miR-3559-3p	2.70E-03	3.39E-02
	miR-132-3p	5.55E-05	4.22E-04
	miR-455-5p	2.09E-03	2.87E-02
	miR-221-3p	8.85E-07	1.12E-05
	miR-34c-5p	2.22E-05	1.68E-03
**Down-regulated miRNAs**			
	miR-148b-3p	1.58E-03	2.61E-02
	miR-144-3p	2.78E-04	1.05E-02
	miR-27a-3p	8.42E-04	2.03E-02
	miR-326-3p	9.42E-04	2.03E-02
	miR-186-5p	1.08E-03	2.03E-02
	miR-196b-5p	3.36E-05	1.69E-02
	miR-206-3p	1.73E-03	2.61E-02
	miR-138-2-5p	5.52E-03	2.27E-02

### Prediction of potential targets of the miRNAs and pathway analysis

**Fig 4:**
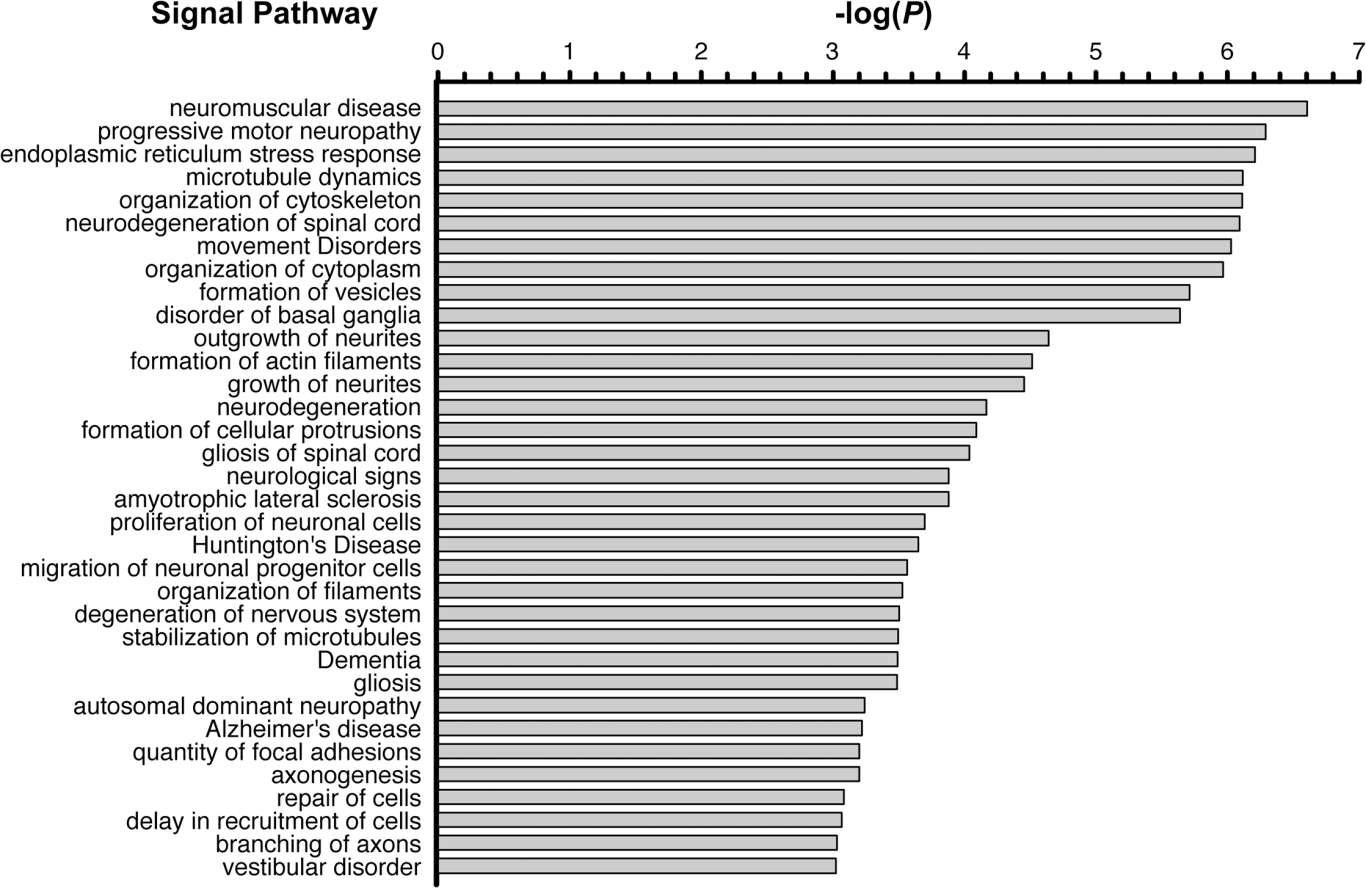
Ingenuity Pathway Analysis of the differentially expressed up-regulated and down-regulated miRNA predicted targets. The listed pathways were from the category of “nervous system development and function” and sorted by *p*-value. Only rows having 3 or more molecules were shown.

To obtain insights into the functional pathways potentially regulated by the miRNAs that showed at least a mean fold-change of 2 following nerve injury, potential downstream target mRNAs were predicted by prediction algorithms such as Targetscan and miRanda. We focused on the seven most up-regulated miRNAs and eight most down-regulated miRNAs along the time series, compared to that of the control (Table 1). The potential target mRNAs commonly predicted by these algorithms were further overlapped with 2,924 transcripts currently known to be localized into distal axons of DRG neurons [27]. Integrating the results from these strategies will help in reducing false positives. We got 78 and 136 intersected different target mRNAs for the significantly up-regulated and down-regulated miRNAs, respectively (S2 Table). The overlapping set of 214 transcripts was first subjected to the Gene Ontology (GO) enrichment analysis to explore a common descriptive framework and functional annotation of the gene sets. As shown in S3 Table, we found that the most GO functions that were significantly enriched by the predicted axonal targets of the miRNAs responding to nerve injury were not simply a direct result of neural regenerative processes, despite the strong association. To further characterize the functional significance of the differentially regulated miRNAs in the nervous system, we next performed Ingenuity Pathway Analysis (IPA), and identified neurological functions that were significantly associated with nerve injury. A total of 34 neurological functions were considered to be significantly changed (*p*<0.05) (Fig 4), showing that miRNAs affect numerous neurological functions involved in ER stress response, cytoskeleton dynamics, vesicle formation, neurite regrowth, and neurodegeneration. Based on these computed bioinformatics analyses, we found that multiple biological and neurological functions were highly associated with alterations of miRNA levels present in nerves during regeneration.

Only a few other studies assessed altered miRNA levels in nervous system relative to uninjured neuron. Yu et al. [26] performed miRNA profiling of the sciatic nerve from the injured sciatic nerve and DRG neurons. Similar to our studies, they also found significantly up-regulated and down-regulated miRNAs both in cell body and proximal stumps of the nerve. However, 36 of 225 (16%) differentially altered miRNAs found in sciatic nerve following injury did not overlap with those present in cell body. Surprisingly, only 3 of these 36 previously identified miRNAs found in sciatic nerve were detected in our studies, suggesting a possibility of non-neuronal cell contamination during RNA extraction from sciatic nerve in the previous study. Contamination from Schwann and other non-neuronal cells is always a concern in cell type-specific gene profiling studies, although there is still a possibility that some of these miRNAs differentially localized in the axon and our analysis here was able to detect only a portion of these. This is particularly important when the gene(s) of interest are at relatively lower levels in the targeted sample than in surrounding cells with a relatively high expression level [30, 31]. Depletion, or at least minimization, of non-neuronal contamination in the axoplasm preparations from sciatic nerve is necessary to study the genes that are present at much lower levels. Furthermore, as shown in S4 Table, 28 of 63 differentially altered axon-specific miRNAs in response to nerve injury detected in our studies did not overlap with differentially expressed miRNAs in the sciatic nerve as previously reported [26]. Therefore, we concluded that, in addition to confirming the findings of altered levels of miRNAs in the sciatic nerve responding to injury, we identified 28 new axon-specific miRNAs whose levels were significantly changed in the injured sciatic nerve relative to the uninjured nerve (S4 Table).

## Supporting Information

S1 Table Annotated rat miRNAs. (XLSX)

S2 Table Predicted axonal targets of miRNAs. (XLSX)

S3 Table Most significant GO functions for the targets of the 15 most altered mRNAs. (DOCX)

S4 Table New Axon-specific miRNAs. (XLSX)
