# Supplementary material for: Dynamic Change and Target Prediction of Axon-Specific MicroRNAs in Regenerating Sciatic Nerve
Source: PLoS One. 2015 Sep 2;10(9):e0137461. doi: 10.1371/journal.pone.0137461 (PMC4557935; doi:10.1371/journal.pone.0137461)
Supplement: S3 Table — (DOCX) [file pone.0137461.s003.docx]

**Supplement Table S3.** The most significant GO functions for the targets of the 15 most altered mRNAs

| GO ID | GO terms | *P* value | # of genes associated |
| --- | --- | --- | --- |
| GO:0050789 | regulation of biological process | 9.71E-14 | 110 |
| GO:0050794 | regulation of cellular process | 1.66E-13 | 105 |
| GO:0065007 | biological regulation | 4.57E-12 | 112 |
| GO:0006950 | response to stress | 8.44E-10 | 47 |
| GO:0032502 | developmental process | 2.12E-09 | 63 |
| GO:0048856 | anatomical structure development | 3.69E-09 | 56 |
| GO:0009987 | cellular process | 4.34E-09 | 138 |
| GO:0048518 | positive regulation of biological process | 5.25E-09 | 54 |
| GO:0033036 | macromolecule localization | 5.95E-09 | 33 |
| GO:0010941 | regulation of cell death | 8.18E-09 | 30 |
| GO:0015031 | protein transport | 9.53E-09 | 26 |
| GO:0045184 | establishment of protein localization | 1.35E-08 | 26 |
| GO:0042981 | regulation of apoptosis | 1.46E-08 | 29 |
| GO:0043067 | regulation of programmed cell death | 1.92E-08 | 29 |
| GO:0048731 | system development | 2.86E-08 | 51 |
| GO:0048522 | positive regulation of cellular process | 3.04E-08 | 49 |
| GO:0007399 | nervous system development | 4.37E-08 | 33 |
| GO:0007275 | multicellular organismal development | 4.68E-08 | 56 |
| GO:0006916 | anti-apoptosis | 9.01E-08 | 12 |
| GO:0008104 | protein localization | 1.97E-07 | 27 |
| GO:0051789 | response to protein stimulus | 2.70E-07 | 11 |
| GO:0019222 | regulation of metabolic process | 3.15E-07 | 64 |
| GO:0007049 | cell cycle | 4.92E-07 | 21 |
| GO:0060548 | negative regulation of cell death | 1.03E-06 | 17 |
| GO:0016043 | cellular component organization | 1.66E-06 | 48 |
| GO:0010033 | response to organic substance | 1.95E-06 | 34 |
| GO:0080090 | regulation of primary metabolic process | 2.17E-06 | 57 |
| GO:0033554 | cellular response to stress | 2.41E-06 | 20 |
| GO:0043066 | negative regulation of apoptosis | 2.47E-06 | 16 |
| GO:0043069 | negative regulation of programmed cell death | 2.89E-06 | 16 |
| GO:0006996 | organelle organization | 3.49E-06 | 31 |
| GO:0048699 | generation of neurons | 3.58E-06 | 21 |
| GO:0060255 | regulation of macromolecule metabolic process | 4.03E-06 | 54 |
| GO:0031323 | regulation of cellular metabolic process | 4.50E-06 | 58 |
| GO:0048869 | cellular developmental process | 4.65E-06 | 36 |
| GO:0046907 | intracellular transport | 6.06E-06 | 20 |
| GO:0030154 | cell differentiation | 6.85E-06 | 35 |
| GO:0009653 | anatomical structure morphogenesis | 6.90E-06 | 28 |
| GO:0006986 | response to unfolded protein | 7.23E-06 | 6 |
| GO:0048523 | negative regulation of cellular process | 1.09E-05 | 38 |
| GO:0051234 | establishment of localization | 1.11E-05 | 49 |
| GO:0051179 | localization | 1.15E-05 | 54 |
| GO:0022008 | neurogenesis | 1.33E-05 | 21 |
| GO:0032989 | cellular component morphogenesis | 1.57E-05 | 14 |
| GO:006810 | transport | 1.68E-05 | 48 |
| GO:0051649 | establishment of localization in cell | 1.69E-05 | 23 |
| GO:0023034 | intracellular signaling pathway | 1.74E-05 | 28 |
| GO:0051171 | regulation of nitrogen compound metabolic process | 2.03E-05 | 47 |
| GO:0010468 | regulation of gene expression | 2.67E-05 | 45 |
| GO:0051716 | cellular response to stimulus | 2.71E-05 | 26 |
| GO:0065009 | regulation of molecular function | 2.87E-05 | 26 |
| GO:0051641 | cellular localization | 3.41E-05 | 24 |
| GO:0050790 | regulation of catalytic activity | 4.54E-05 | 23 |
| GO:0009889 | regulation of biosynthetic process | 5.28E-05 | 46 |
| GO:0009605 | response to external stimulus | 5.74E-05 | 19 |
| GO:0009893 | positive regulation of metabolic process | 6.04E-05 | 26 |
| GO:0009991 | response to extracellular stimulus | 6.25E-05 | 15 |
| GO:0031325 | positive regulation of cellular metabolic process | 6.79E-05 | 25 |
| GO:0019219 | regulation of nucleobase, nucleoside, nucleotide and nucleic acid metabolic process | 6.88E-05 | 45 |
| GO:0030182 | neuron differentiation | 7.69E-05 | 15 |
| GO:0009719 | response to endogenous stimulus | 7.72E-05 | 22 |
| GO:0031326 | regulation of cellular biosynthetic process | 7.73E-05 | 45 |
| GO:0000902 | cell morphogenesis | 8.07E-05 | 12 |
| GO:0009628 | response to abiotic stimulus | 8.50E-05 | 16 |
